# A Soluble Pyrophosphatase Is Essential to Oogenesis and Is Required for Polyphosphate Metabolism in the Red Flour Beetle (*Tribolium castaneum*)

**DOI:** 10.3390/ijms16046631

**Published:** 2015-03-24

**Authors:** Klébea Carvalho, Lupis Ribeiro, Jorge Moraes, José Roberto da Silva, Evenilton P. Costa, Jackson Souza-Menezes, Carlos Logullo, Rodrigo Nunes da Fonseca, Eldo Campos

**Affiliations:** 1 Laboratory of Integrated Biochemistry—Hatisaburo Masuda, Universidade Federal do Rio de Janeiro, Núcleo em Ecologia e Desenvolvimento Sócio Ambiental de Macaé, Avenida São José do Barreto, 764, São José do Barreto, Macaé, RJ CEP 27965-045, Brazil; E-Mails: klebeacarvalho@gmail.com (K.C.); lupisribeiro@gmail.com (L.R.); jorgemoraes@bioqmed.ufrj.br (J.M.); beto_cfrio@yahoo.com.br (J.R.S.); jacksonmenezes@gmail.com (J.S.-M.); rodrigo.nunes.da.fonseca@gmail.com (R.N.F.); 2 Nacional Institute of Science and Technology—Molecular Entomology, Rio de Janeiro, RJ CEP 21941-590, Brazil; E-Mail: logullo@uenf.br; 3 Laboratory of Chemistry and Function of Proteins and Peptides and Unity of Animal Experimentation, Biotecnology and Bioscience Center, Universidade Estadual do Norte Fluminense, Avenida Alberto Lamego, 2000, Horto, Campos dos Goytacazes, RJ CEP 28015-620, Brazil; E-Mail: eveniltonpessoa@yahoo.com.br

**Keywords:** polyphosphate, pyrophosphatase, oogenesis, embryogenesis, insect

## Abstract

Polyphosphates have been found in all cell types examined to date and play diverse roles depending on the cell type. In eukaryotic organisms, polyphosphates have been mainly investigated in mammalian cells with few studies on insects. Some studies have demonstrated that a pyrophosphatase regulates polyphosphate metabolism, and most of them were performed on trypanosomatids. Here, we investigated the effects of *sPPase* gene knocked down in oogenesis and polyphosphate metabolism in the red flour beetle (*Tribolium castaneum*) A single *sPPase* gene was identified in insect genome and is maternally provided at the mRNA level and not restricted to any embryonic or extraembryonic region during embryogenesis. After injection of *Tc-sPPase* dsRNA, female survival was reduced to 15% of the control (dsNeo RNA), and egg laying was completely impaired. The morphological analysis by nuclear DAPI staining of the ovarioles in *Tc-sPPase* dsRNA-injected females showed that the ovariole number is diminished, degenerated oocytes can be observed, and germarium is reduced. The polyphosphate level was increased in cytoplasmic and nuclear fractions in *Tc-sPPase* RNAi; Concomitantly, the exopolyphosphatase activity decreased in both fractions. Altogether, these data suggest a role for *sPPase* in the regulation on polyphosphate metabolism in insects and provide evidence that *Tc-sPPase* is essential to oogenesis.

## 1. Introduction

Inorganic polyphosphates (poly P) are long chains of a few to several hundred phosphate residues linked by phosphoanhydride bonds. Taking into consideration their significance in all living organisms, inorganic polyphosphates may be separated into two groups—Namely, pyrophosphate and high-molecular-weight poly Ps—Which contain three to several hundred phosphate residues in one molecule [1]. Polyphosphates have been found in all cell types examined to date and play diverse roles depending on the cell type. The biological roles played by polyphosphates have been most extensively studied in prokaryotes and unicellular eukaryotes [2,3]. In higher eukaryotes, poly P have not been extensively investigated, although a role in the activation of Tor kinase [4], blood coagulation [5], bone tissue development [6], and apoptosis [7] have been reported.

Poly P metabolism can be specific in different cellular compartments [3,8,9,10]. In nuclei, for microorganisms, it has been reported that poly P control gene expression [11] is a potent inhibitor of the degradosome-dependent degradation of mRNA [12] and a promoter of ribosomal [13] and nucleoid protein degradation by activating Lon protease [14]; It also assists in the fidelity of protein translation by its interaction with ribosomes [15]. In yeast, poly P interact and inhibit poly(A) polymerase activities [16,17]. In mammalian cells, poly P accumulates and regulates myeloma proliferation [18].

To understand the relationship between poly P metabolism and its cellular distribution, studies on poly P composition and on the enzymes required for its metabolism were performed [10,19]. Soluble inorganic pyrophosphatases (*sPPases*, EC 3.6.1.1) catalyze the hydrolysis of inorganic pyrophosphate (PPi), which is formed mainly as a product of many biosynthetic pathways, including oligosaccharide and fatty acid synthesis, tRNA charging/amino acid activation, and polynucleotide synthesis [20]. In nuclei, pyrophosphate is generated as a metabolite of replication, transcription, and DNA repair. During nucleic acid polymerization, the incorporation of a nucleoside in the growing chain with the concomitant liberation of pyrophosphate is a readily reversible reaction, and the hydrolysis of pyrophosphate by *sPPase* eliminates metabolite inhibition, therefore, shifting the reaction equilibrium in favor of polymerization [21]. Polyphosphate hydrolysis is catalyzed by exo- and endopolyphosphatases [3]. Exopolyphosphatases (PPX—Polyphosphate-phosphohydrolases; EC 3.6.1.11) splits P_i_ off the end of a poly P chain and are considered as the central regulatory enzymes in poly P metabolism [9,22].

To our knowledge, a few studies have demonstrated that a pyrophosphatase regulates polyphosphate metabolism, and most of them were performed on trypanosomatids [19,22,23]. Here, we investigated the polyphosphate metabolism in the red flour beetle, *Tribolium castaneum*, which is a common pest that has emerged as an excellent model system for studying development and metabolism in insects [24,25,26]. This beetle is amenable to gene knockdown by RNA interference (RNAi). Any gene can be knocked down at any stage in all tissues upon injection of double-stranded RNA (dsRNA) [27,28], have its genome sequenced [29], and have developed mutants [30].

In the present work, we analyzed the effects of the *sPPase* gene knocked down in oogenesis and polyphosphate metabolism. Our results show that *PPase* is essential to oogenesis and plays a role in poly P metabolism in cytoplasmic and nuclei fractions.

## 2. Results and Discussion

Thus far, poly P metabolism has been mainly investigated in mammalian cells [5,31,32,33], with few studies in insects [34,35,36]. Since all previous analyses of pyrophosphatase regulating polyphosphate metabolism were performed in trypanosomatids, we sought to investigate the enzymes involved in the red flour beetle (*Tribolium castaneum*).

### 2.1. A Single Soluble Inorganic Pyrophosphatases (sPPases) Can Be Identified in Tribolium castaneum and Other Insect Genomes

**Figure 1:**
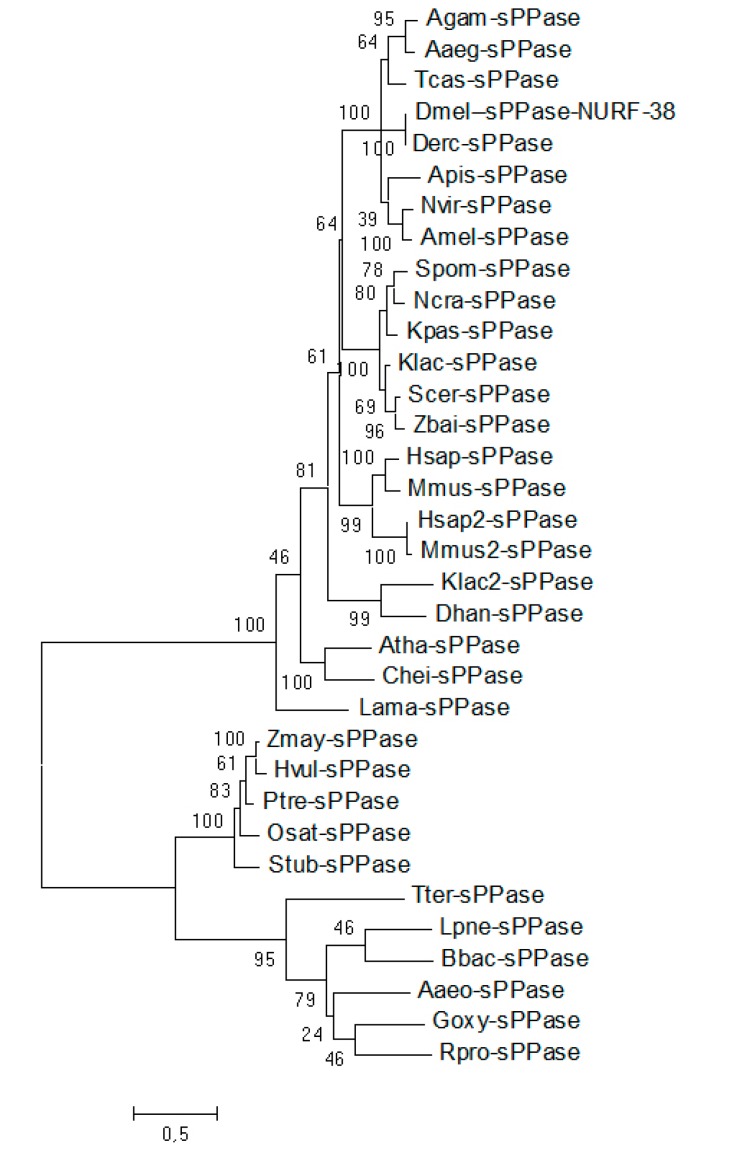
A single *sPPAse* is present in insect genomes. Gene bank accession numbers are as follows: XP 558852.4 *Anopheles gambiae* (*Agam-sPPase*), ABF18311.1 *Aedes aegypti* (*Aaeg-sPPase*), EEZ98942.1 TC004566 *Tribolium castaneum* (*Tcas-sPPase*), AAC97112.1 NURF-38 *Drosophila melanogaster* (*Dmel-sPPase-NURF-38*), XP 001976623.1 *Drosophila erecta* (*Derc-sPPase*), XP 008183751.1 *Acyrthosiphon pisum* (*Apis-sPPase*), XP 001604166.1 *Nasonia vitripennis* (*Nvit-sPPase*), XP 003249382.1 *Apis mellifera* (*Amel-sPPase*), P19117 *Schizosaccharomyces pombe* (*Spom-sPPase*), Q6MVH7 *Neurospora crassa* (*Ncra-sPPase*), O13505 *Komagataella pastoris* (*Kpas-sPPase*), P13998 *Kluyveromyces lactis* (*Klac-sPPase*), P00817 *Saccharomyces cerevisiae* (*Scer-sPPase*), Q9C0T9 *Zygosaccharomyces bailii* (*Zbai-sPPase*), Q9H2U2 *Homo sapiens* (*Hsap-sPPase*), Q91VM9 *Mus musculus* (*Mmus-sPPase*), Q15181 *Homo sapiens* (*Hsap2-sPPase*), Q9D819 *Mus musculus* (*Mmus2-sPPase*), Q6CNP0 *Kluyveromyces lactis* (*Klac2-sPPase*), Q6BLR8 *Debaryomyces hansenii* (*Dhan-sPPase*), Q9LXC9 *Arabidopsis thaliana* (*Atha-sPPase*), Q93Y52 *Chlamydomonas reinhardtii* (*Chei-sPPase*), Q7Z031 *Leishmania amazonensis* (*Lama-sPPase*), O48556 *Zea mays* (*Zmay-sPPase*), O23979 *Hordeum vulgare* (*Hvul-sPPase*), Q9SWI0 *Populus tremula* (*Ptre-sPPase*), Q0DYB1 *Oryza sativa* (*Osat-sPPase*), O49949 *Solanum tuberosum* (*Stub-sPPase*), P38576 *Thermus thermophiles* (*Tter-sPPase*), O34955 *Legionella pneumophila* (*Lpne-sPPase*), P51064 *Bartonella bacilliformis* (*Bbac-sPPase*), O67501 *Aquifex aeolicus* (*Aaeo-sPPase*), O05545 *Gluconobacter oxydans* (*Goxy-sPPase*), Q9ZCW5 *Rickettsia prowazekii* (*Rpro-sPPase*). Substitution model used LG + G + I with 100 bootstraps.

BLAST searches using the previously reported nuclear, cytosolic, or acidocalcisomal *sPPases* [22] against the *Tribolium castaneum* genome lead to a single ortholog, the *Tc-004566* gene. Other insect genomes also display a single locus belonging to *sPPases* (Figure 1), suggesting that the previously reported paralogs with distinct functions in trypanosomatids or humans [22] probably arose due to independent duplication events.

**Figure 2:**
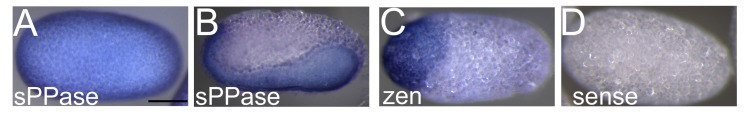
*Tc-pyrophosphatase* (*Tc-sPPase*) is maternally provided and expressed during embryogenesis. (**A**) Freshly laid *Tribolium castaneum* egg showing *Tc-sPPase* expression; (**B**) *Tc-sPPase* expression during germ band elongation; (**C**) *Tc-zen* expression at the serosa during blastoderm differentiation; and (**D**) *In situ* hybridization using a *Tc-sPPase* probe sense control. Scale bar = 200 µm.

Since the *T. castaneum* genome displays a single *sPPase* ortholog, we sought to analyze its expression by *in situ* hybridization during embryogenesis. *Tc-sPPase* (*Tc-004566*) is maternally provided at the mRNA level and not restricted to any embryonic or extraembryonic region during embryogenesis (Figure 2A), probably due to the requirement of energy sources and phosphates for early proliferation stages of embryogenesis. During germ band elongation, when the embryo can be readily identified, the expression can be detected in the embryo and in the extraembryonic tissue (Figure 2B). In contrast, the *Tc-zen* gene is specifically expressed at the extraembryonic serosal region (Figure 2C), as previously described [37].

### 2.2. pRNAi Analysis Shows that Tc-sPPase Is Essential to Oogenesis and Regulates Poly P Metabolism

To investigate if *Tc-sPPase* is important for oogenesis and how its absence would affect poly P metabolism, parental RNAi (pRNAi) was performed as previously described for several other genes in this species [38,39]. In all experiments, we injected the unrelated dsRNA neomycin as a negative control in a separate batch of females. These neomycin dsRNA females laid the normal amount of eggs, which hatched as larvae, indicating that the injection of unrelated dsRNA had no effect on *T. castaneum* development.

**Figure 3:**
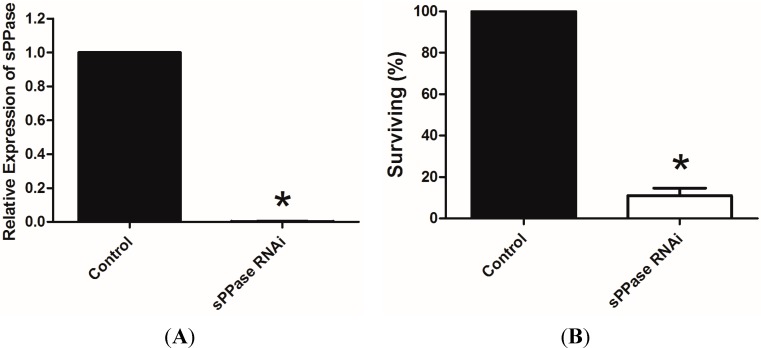

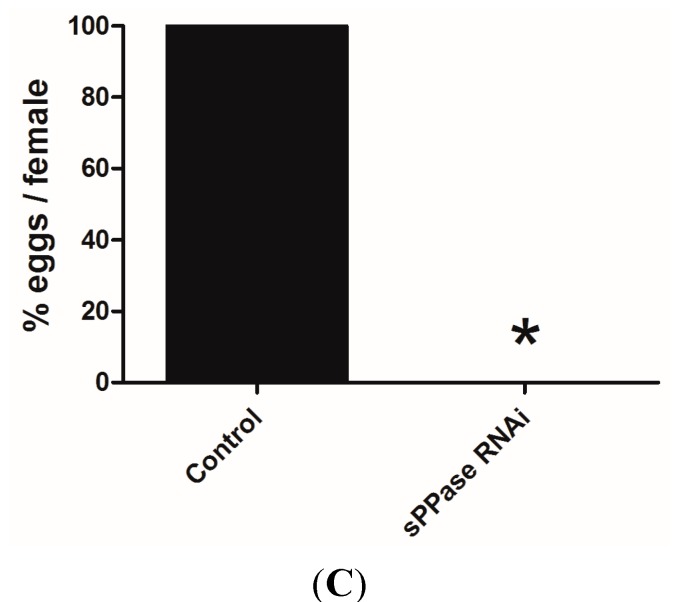
(**A**) Comparison of *Tc-sPPase* in control (dsNeo RNA) and *sPPase* dsRNA injected adults. Normalized levels to *Tc-Rps3* expression as previously described [26]; (**B**) Adult survival in control and *sPPase* dsRNA injected beetles (%); and (**C**) Egg laying of control and *sPPase* dsRNA–injected beetles (%). Asterisk indicates that the difference between the two groups is statistically significant (*p* < 0.05).

We analyzed *Tc-sPPase* expression in control (dsNeo) and *Tc-sPPase* RNAi by real-time PCR. Injection of *Tc-sPPase* dsRNA (1 µg/µL) almost completely inhibited its expression when compared to the control (Figure 3A), confirming that *Tc-sPPase* transcription was affected. After injection of *Tc-sPPase* dsRNA, female survival was also reduced to 15% of the control (Figure 3B), and egg laying was completely impaired (Figure 3C), suggesting that *Tc-sPPase* is essential to oogenesis.

**Figure 4:**
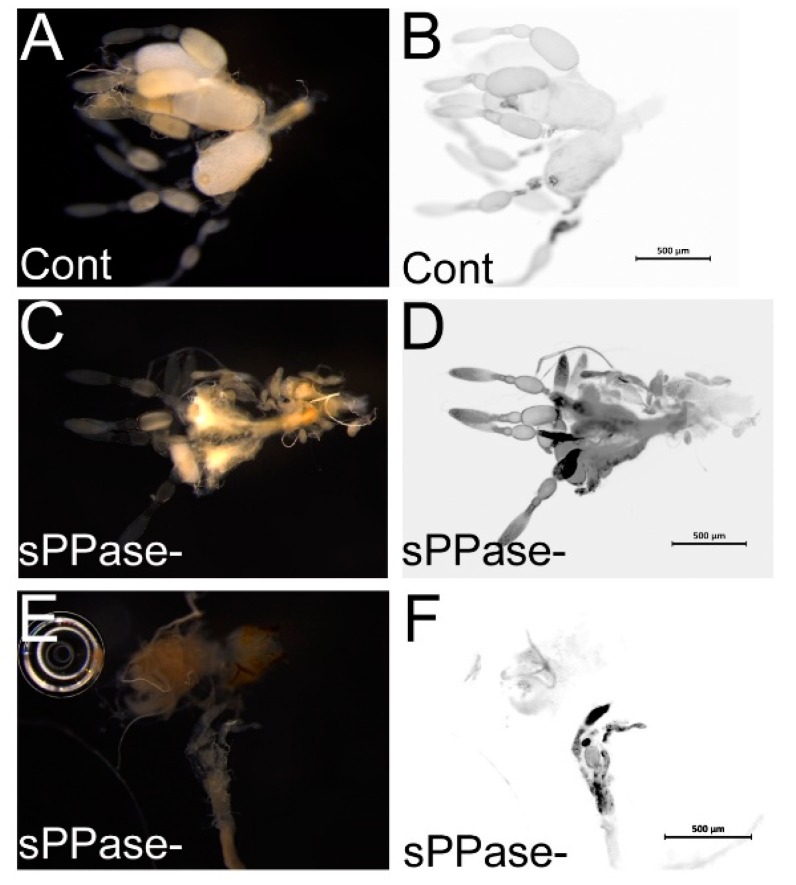
(**A**) Control dsNeoRNA ovarioles; (**B**) Nuclear DAPI (4',6-diamidino-2-phenylindole) staining of the ovary as in (**A**); (**C**) *sPPase* dsRNA–injected ovarioles; (**D**) Nuclear DAPI staining of the ovary in (**C**); (**E**) *sPPase* dsRNA–injected ovarioles; and (**F**) Nuclear DAPI staining of the ovary in (**E**).

These results stimulated the analysis of the morphology of *Tc-sPPase* RNAi ovaries. The morphological analysis by nuclear DAPI staining of the ovarioles of control and *Tc-sPPase* dsRNA–injected females showed clear differences (Figure 4). *T. castaneum* control ovaries display several tubelike projections, the ovarioles (e.g., [40]), which contains oocytes in different stages of maturation. In control ovaries, larger eggs are present in the distal part of the ovariole (Figure 4A,B). However, after *Tc-sPPase* RNAi injection, the ovariole number is diminished, degenerated oocytes can be observed, and germarium is reduced (Figure 4C,D). In strong *Tc-sPPase* RNAi (20%), a complete degradation of the ovarioles was observed (Figure 4E,F). This result reinforces the essential role of *Tc-sPPase* in oogenesis.

**Figure 5:**
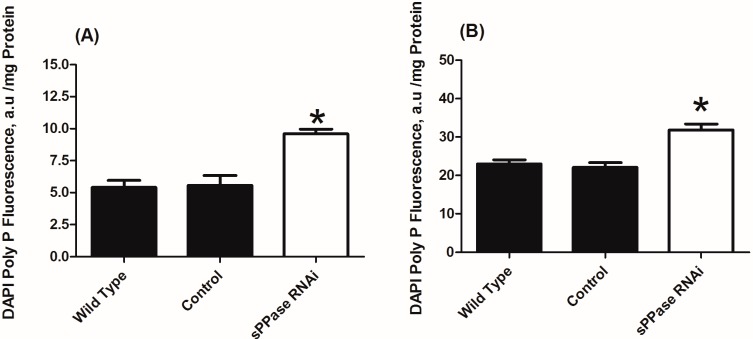

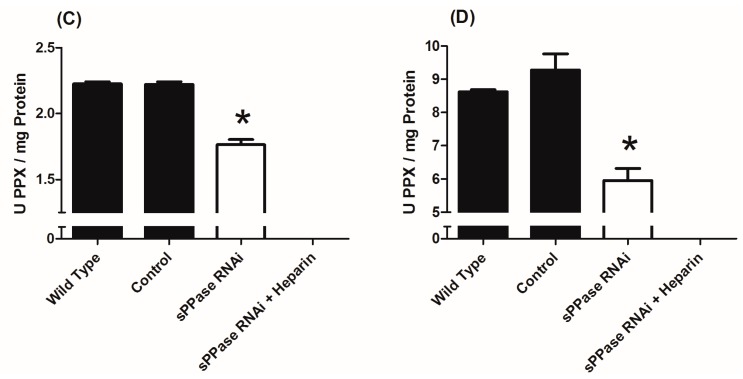
(**A**) Cytoplasm poly P fluorescence (DAPI) of wild-type, control (dsNeo RNA), and *sPPase* RNAi beetles; (**B**) Nuclear poly P fluorescence (DAPI) of wild-type, control (dsNeo RNA), and *sPPase* RNAi beetles; (**C**) Cytoplasmic PPX activity in wild-type, control (dsNeo RNA), and *PPX* activity in the presence and absence of heparin; and (**D**) Nuclear PPX activity in wild-type, control (dsNeo RNA), and *PPX* activity in the presence and absence of heparin. Data are the mean ± S.E. (standard error) of three independent experiments, in triplicate. * *p* < 0.05.

To evaluate the effect of *Tc-sPPase* RNAi in poly P metabolism, we determined in cytoplasmic and nuclear fractions the poly P content and PPX activity of wild-type, control (dsNeo RNA) and *Tc-sPPase* dsRNA injected-females. The poly P level was increased in cytoplasmic and nuclear fractions by factors of 2 and 1.5, respectively, in *Tc-sPPase* RNAi when compared to control (Figure 5A,B). Concomitantly, PPX activity decreased to levels of about 70% of the control in both fractions. Heparin, a PPX inhibitor, was used as control and completely abolished PPX activity (Figure 5C,D). These data suggest a role for *sPPase* in the regulation of poly P metabolism. Previous studies have found that a pyrophosphatase plays a key role in poly P metabolism as an alternative to exopolyphosphatase activity in eukaryotic cells, being required in poly P metabolism, metacyclogenesis, and virulence in mice [19,22]. However, both studies were performed in trypanosomatids, to the best of our knowledge; This is the first study to demonstrate that the pyrophosphatase plays a key role in poly P metabolism in an insect.

The sequence analysis of *Tc-PPase* revealed that it is homologue to NURF-38 from *Drosophila melanogaster* [21]. The *Drosophila* NURF is a component of several macromolecular complexes involved in nucleosome dynamics or histone metabolism, and both the NURF-38 protein and the purified NURF complex were found to possess inorganic *sPPase* activity [21]. We propose that *sPPase* gene knockdown may disturb gene expression in two ways: By NURF complex alteration and/or by nuclear poly P increase. Nuclear poly P may interact with DNA-histone binding in chromatin, and this binding has been shown to inhibit the activity of some nuclear enzymes, including topoisomerases [41]. In general, we propose that the extensive morphological effects observed during oogenesis could be caused by disturbance of gene expression required for embryo formation.

## 3. Experimental Section

### 3.1. Tribolium castaneum Strains

San Bernardino beetles are reared at 30 °C in wheat flour supplemented with 5% dried yeast. The beetles were maintained inside plastic boxes of approximately 15 cm × 15 cm with humidity between 40% and 80% as previously described [42].

### 3.2. Sequence Analysis

*sPPases* were identified in the *Tribolium castaneum* genome [29] using the BLAST program (NCBI). Amino acid alignment and analysis of *sPPase* similarity for selected species were performed using the ClustalW multiple sequence alignment program (http://www.ebi.ac.uk/clustalw). Maximum likelihood phylogenies were generated with PhyML [43]. Trees were edited in MEGA5.05 [44]. The accession numbers of the various sequences used in that study are described in the figure legend.

### 3.3. Primer Design, in Situ Hybridization and RNAi

Primer sequences containing adaptor sequences (lowercase letters) were designed for *in situ* hybridization and dsRNA synthesis with the help of Primer3 (www.ncbi.nlm.nih.gov/tools/primer-blast/) ggccgcggTCACGATGTCCCTTTGGTGG (Forward) and cccggggcGACCTTTTGCGGTTGCGAAT (Reverse) and the PCR product size was 736 bp. A second primer pair led to identical results (data not shown). Double-stranded RNA (dsRNA) was synthesized using T7 MEGAScript (Ambion, Austin, TX, USA), purified and injected in adult females as previously described [45]. *In situ* hybridization was performed using digoxigenin-labeled RNA probes and revealed with alkaline phosphatase chromogenic substrate BM Purple (Roche, Indianapolis, IN, USA). The one color *in situ* protocol for *Tribolium* was done as described [46] followed by nuclear DAPI staining (4,6-diamidino-2-phenylindole) before documentation. A sense probe was included during *in situ* hybridization experiments and did not show any specific staining.

### 3.4. Real-Time PCR

Total RNA was isolated from 100 mg of eggs using TrizolH (Invitrogen, Carlsbad, CA, USA) according to the manufacturer’s instructions. First-strand complementary DNA (cDNA) was synthesized using Superscript III reverse transcriptase (Invitrogen), and real-time PCR analysis using SYBR green-based detection was performed. For real-time PCR, the following primer pair was used: Fwd-CGCTTTTAATGGCGAAGCGA and Rev-AGGAAATGCCCTTGGCATCA leading to an amplicon of 113 bp. The reactions were carried out in triplicate, and melting curves were examined to ensure single products. The results were quantified using the “ΔΔ*C*_t_” method and normalized to rps3 transcript levels and to control genotypes [47]. Data shown are averages and standard deviations from at least three independent experiments.

### 3.5. Cell Fractionation

Adult female was used to obtain the cytoplasmic and nuclear fractions. The adult female were homogenized in 1 mL of an isolation buffer containing 0.5 M sucrose, 50 mM Tris-HCl (pH 7.4), 100 mM leupeptin, and 100 nM pepstatin. The homogenate was centrifuged at 500× *g* for five minutes to remove unbroken cell and other debris. The supernatant was carefully removed and centrifuged at 2000× *g* for 10 min to yield a nuclear pellet. Then, the supernatant was submitted to another centrifugation at 100,000× *g* for one hour to obtain the cytoplasmic fraction in the supernatant. The nuclear pellet was rinsed with cold isolation buffer and resuspended.

### 3.6. Exopolyphosphatase Activity

The reaction mixture consisted of 50 mM Tris-HCl buffer (pH 7.5), 5 mM MgCl_2_, and 5 mM polyP_15_, as the substrate. The reaction was carried out at 30 °C for 15 min. The activity was measured spectrophotometrically (UV Mini 1240, Shimadzu, Tokyo, Japan) by determining the rate of the Pi formed during the reaction as described by [48]. The measurements of absorbance at 750 nm were performed after 15 min. Protein concentration was measured as described by [49], using bovine serum albumin as standard. The enzyme amount liberating 1 μmol of Pi per one minute was defined as one unit of enzyme activity (U).

### 3.7. Poly P Extraction and Quantification

The cytoplasmic and nuclear fractions were mixed with equal volume of acid phenol/chloroform, pH 4. The samples were vortexed for 5 min at 4 °C, followed by centrifugation at 1500× *g* for 5 min at 4 °C. The water phase was transferred to a new tube and subjected to chloroform extraction with the equal volume of chloroform to remove traces of organic solvents from the water phase. Poly P was precipitated from the water phase by adding 2.5 volumes of ethanol, followed by overnight incubation at −20 °C. The water-ethanol mixture was centrifuged for 10 min at 10,000× *g*. The resulting pellet containing poly P was resuspended in 50 μL of a buffer (0.1% SDS, 1 mM EDTA, and 10 mM Tris-HCl, pH 7.4) treated with RNase and DNase to remove nucleic acid contamination [34,35,50].

DAPI fluorescence was measured using a spectrofluorometer (Cary Eclipse, Agilent Technologies, Santa Clara, CA, USA). A Teflon stir bar was used to continuously mix the sample during fluorescence measurements. The nuclear or cytoplasmic fractions were incubated with 20 mg/mL DAPI for 30 min at room temperature. The fluorescence level at 550 nm obtained using 415 nm as the excitation wavelength was used to estimate the variation of poly P. PolyP_75_ (150 µg) was used to normalize the values obtained. The values of poly P were expressed as a function of the relative fluorescence signal obtained from fractions and polyP_75_ [35,51].

### 3.8. Statistical Analysis

Comparisons between groups were made by the non-paired Student’s *t* test and ANOVA One-way analysis of variance, using GraphPad Prism. For all tests, a difference of *p* < 0.05 was considered to be significant.
